# A Robust Terrain Aided Navigation Using the Rao-Blackwellized Particle Filter Trained by Long Short-Term Memory Networks

**DOI:** 10.3390/s18092886

**Published:** 2018-08-31

**Authors:** Jungshin Lee, Hyochoong Bang

**Affiliations:** Department of Aerospace Engineering, KAIST, 291 Daehak-ro, Yuseong-gu, Daejeon 34141, Korea; skydoumi@kaist.ac.kr

**Keywords:** terrain-aided navigation (TAN), Rao-Blackwellized particle filter (RBPF), long short-term memory (LSTM), terrain validity check, digital elevation model (DEM), inertial navigation system (INS)

## Abstract

Terrain-aided navigation (TAN) is a technology that estimates the position of the vehicle by comparing the altitude measured by an altimeter and height from the digital elevation model (DEM). The particle filter (PF)-based TAN has been commonly used to obtain stable real-time navigation solutions in cases where the unmanned aerial vehicle (UAV) operates at a high altitude. Even though TAN performs well on rough and unique terrains, its performance degrades in flat and repetitive terrains. In particular, in the case of PF-based TAN, there has been no verified technique for deciding its terrain validity. Therefore, this study designed a Rao-Blackwellized PF (RBPF)-based TAN, used long short-term memory (LSTM) networks to endure flat and repetitive terrains, and trained the noise covariances and measurement model of RBPF. LSTM is a modified recurrent neural network (RNN), which is an artificial neural network that recognizes patterns from time series data. Using this, this study tuned the noise covariances and measurement model of RBPF to minimize the navigation errors in various flight trajectories. This paper designed a TAN algorithm based on combining RBPF and LSTM and confirmed that it can enable a more precise navigation performance than conventional RBPF based TAN through simulations.

## 1. Introduction

Aircraft safety requires highly reliable navigation information. Traditionally, the inertial navigation system and global positioning system (INS/GPS) integrated navigation algorithm has been widely used [1]. However, GPS cannot operate independently and is also vulnerable to jamming. To overcome such weakness, the terrain-aided navigation (TAN) techniques can be used. TAN is a navigation technology that estimates the aircraft’s precise position by comparing the altitude measured by an altimeter with the uploaded digital elevation data (DEM). To acquire precise position information using TAN, nonlinear estimation problems must be solved in real-time. The extended Kalman filter (EKF)-based TAN algorithms have solved these problems through regional linearization [2]. However, because of the highly nonlinear characteristics of the terrain, the EKF-based TAN algorithm can diverge due to linearization. Recent studies have suggested that the TAN techniques that use the Bayesian estimate method, such as particle filter (PF) and point mass filter (PMF), can prevent the problem [3,4,5,6,7]. The techniques can be directly applied to nonlinear problems without having to perform linearization, like EKF. When using the Bayesian approach, integration terms are included when the measurements are updated. It’s difficult to calculate the integration terms in real time. PF uses the Monte-Carlo sampling method instead of the integration terms required to normalize the posterior pdf via Bayes’ rule [8]. Increasing the precision of PF requires many particles, and the computation load rapidly increases if the dimension of the state variables increases. As a means for more efficient calculations, there have been studies that applied the Rao-Blackwellization technique where state variables are divided into linear and nonlinear parts [9,10,11]. In this study, the two-dimensional PF was composed of latitude and longitude errors. The altitude bias generates errors in the likelihood calculations of the PF. To compensate for this, a one-dimensional Kalman filter was added. If the three-dimensional PF was composed of latitude, longitude, and altitude errors, more particles are needed to ensure accuracy. This causes the computational complexity. To alleviate this problem, we used the Rao-Blackwellization technique that marginalize the states that vary close to linearly in the dynamics. One Kalman filter was assigned to each particle by marginalization.

TAN for aircraft, UAVs, and missiles is a well-established technique that has been studied for several decades. Recently, there have been active studies in the field of the autonomous underwater vehicles (AUVs) [10,12]. The main differences between TAN for AUVs and aerial vehicle systems are the vehicle dynamics and sensors used to measure the relative position from the vehicle to the terrain [12]. In particular, the AUVs and UAVs systems require high reliability and have to guarantee a stable navigation performance in the GPS-less environment, but most studies about the TAN technique for AUVs and UAVs have focused on rough and unique terrains. Otherwise, there have been studies that represented the stable TAN performance to make a detour around the flat and repetitive terrains through path planning or simultaneous localization and mapping (SLAM) techniques [12,13], but as vehicles have been recently required to perform various missions, even in GPS-less environments, the path planning or SLAM techniques are limited in terms of survivability and reliability. Therefore, in this study, we suggest the robust TAN technique for reliable navigation performance, even in flat or repetitive terrains. In other words, we designed a TAN technique that can perform in flat or repetitive terrain, instead of avoiding these terrains and moving into rough or unique terrains by using the path planning and SLAM techniques.

For the robust TAN, the validity check technique of measurements by terrain roughness and uniqueness is an important technique that determines the navigation performance. The mean squared difference (MSD) and mean absolute difference (MAD) of the height deviation have been widely used for contour matching based TAN [14,15]. As for the bank of Kalman filter (BKF)-based TAN, the validity check technique that uses smoothed weighted residual squared (SWRS) is standard [16]. However, for the TAN that uses Bayesian filters, like PF and PMF, there is no commonly used validity check technique. Therefore, at first, we considered the validity check technique by using mutual information (MI) and residual check logic, which can be applied to PF. In [17], the validity check technique that uses MI was introduced. The MI about the joint probability function of the likelihood and prior probability distribution measures how much the likelihood reduces uncertainty about the prior distribution. Therefore, if the value of MI is positive, the likelihood from generating the measurement can be useful [17]. However, although the measurement error is instantaneously large, using the measurement can be helpful to the PF in some cases. In other words, the current value of MI is not enough to provide robust solutions on certain flight trajectories. Also, the incorrect estimates of PF can cause the validity check logic by using residuals between the measurements and the estimates to malfunction. The validity check technique cannot thoroughly guarantee the reliability and robustness of the TAN.

Next, we considered the method that can control the noise covariance and measurement model to reduce the navigation error. There have been studies that estimated the process noise by modelling the magnitude of the maximum uncertainties or the sufficient statistics of the process and measurement noise parameters [18,19]. However, this method models the magnitude of noise at the moment accurately instead of determining the optimal process and measurement noise for generating stable particles in flat and repetitive terrains. In other words, modeling close to the true magnitude of noise may, at times, degrade the filter performance in flat and repetitive terrains. To solve this problem, this study adopted an improved recurrent neural networks (RNN) method called long short-term memory (LSTM). RNN is an artificial neural network that recognizes patterns from the time series data, which is one of the deep learning techniques that considers current and past input data via inner memory [20]. However, as for the RNN, its error gradient decreases along with back propagation when going back in time, so it is inappropriate to analyzing long time series data patterns. LSTM networks update intermediate memory cells with the sum of values that pass through the input and output gates, so they can deal with longer sequences than RNN, which is composed of only multiplication. Recently, there have been active studies that use the expectation-maximization (EM) algorithm or Bayes rules to efficiently conduct RNN or LSTM training or studies that use LSTM to improve the performance of KF or PF [21,22,23,24,25,26]. These studies are not suitable for providing real-time solutions or they are mostly limited to image recognition. This paper used LSTM networks to train noise covariances and measurement model of RBPF based TAN for improving the navigation performance in flat and repetitive terrains.

Section 2 summarizes the design of the conventional RBPF based TAN. Section 3 introduces the terrain and measurement validity check logic and its application to the designed RBPF-based TAN. Next, the LSTM modules are designed, and an LSTM-RBPF-based TAN is proposed, of which the noise covariances and measurement model are trained by the LSTM modules. Finally, this study determines the model parameters for the proposed LSTM modules using training data and performs Monte Carlo simulations that use evaluation data to verify the proposed design.

## 2. Conventional RBPF Based TAN

As for the EKF-based TAN, there is a high probability of divergence if the nonlinearity of the system or measurement model is too great. Therefore, this study considered PF-based TAN. PF is one of the general Bayesian filters that use global approximation instead of regional linearization [3,8,27]. In this study, the two-dimension PF was composed of latitude and longitude errors. The Bayesian filter was applied to the following TAN system and measurement model:

(1) $$x_{k}=x_{k-1}+u_{k}+w_{k-1},$$

(2) $$y_{k}=h_{k}\left(x_{k}\;\right)+v_{k},$$

$x_{k}={\left[\begin{array}{cc} \delta \phi & \delta \lambda \end{array}\right]}^{T}$ is a two-dimensional state vector composed of latitude and longitude errors at the k-th time. $u_{k}={\left[\begin{array}{cc} v_{n,k} & v_{e,k} \end{array}\right]}^{T}$ and $h_{k}\left(x_{k}\right)$ denote the velocity vector composed of velocities in a northward and eastward direction and terrain elevation from the DEM evaluated at the position, $x_{k}$. $y_{k}$ is the terrain height calculated by the measurements of the IRA and barometer. In the system above, $w_{k}$ is the system white noise that meets $E\left(w_{k}\right)=0$ and $E\left(w_{k}\right)E{\left(w_{k}\right)}^{T}=Q_{k}\Delta t$. Here, $Q_{k}$ is the system process noise covariance, and Δt is the sampling time. In the measurement above, $v_{k}$ is the white measurement noise that meets $E\left(v_{k}\right)=0$ and $E\left(v_{k}\right)E{\left(v_{k}\right)}^{T}=R_{k}$. Here, $R_{k}$ is the measurement noise covariance. The prior pdf is as follows. The prediction step uses the TAN system model (1) to obtain the prior pdf of the state at time step k via the Chapman-Kolmogorov Equation (6):

(3) $$p\left(x_{k}|x_{k-1}\right)=p_{w_{k-1}}\left(x_{k}-x_{k-1}-u_{k}\right),$$

By using the same process above from Equation (2), the likelihood is as follows:

(4) $$p\left(y_{k}|x_{k}\right)=p_{v_{k}}\left(y_{k}-h_{k}\left(x_{k}\right)\right),$$

At time step k, measurement $y_{k}$ becomes available, and this may be used to update the prior pdf via Bayes’ rule [28]. The posterior pdf that uses this is as follows:

(5) $$p\left(x_{k}|Y_{k}\right)=\frac{1}{\alpha_{k}}p_{v_{k}}\left(y_{k}-h_{k}\left(x_{k}\right)\right)p\left(x_{k}|Y_{k-1}\right),$$

Here, $\alpha_{k}=\int^{\text{​}}p_{v_{k}}\left(y_{k}-h_{k}\left(x_{k}\right)\right)p\left(x_{k}|Y_{k}\right)dx_{k}$ and $Y_{k}=\left\{y_{1},y_{2},\;\ldots ,\;y_{k}\right\}$. $\alpha_{k}$ is the parameter that normalizes the posterior pdf. The state variable estimate and covariance that minimize the mean square error are as follows:

(6) $${\hat{x}}_{k|k}=\int^{\text{​}}x_{k}p\left(x_{k}|Y_{k}\right)dx_{k},$$

(7) $${\hat{p}}_{k|k\;}=\int^{\text{​}}\left(x_{k}-{\hat{x}}_{k|k}\right){\left(x_{k}-{\hat{x}}_{k|k}\right)}^{T}p\left(x_{k}|Y_{k}\right)dx_{k},$$

The computational load due to the integral calculation included in the above conditional pdf is large. To alleviate the computational load, the sequential importance sampling-PF (SIS-PF) is generally used. SIS-PF is a technique that implements a sequential Bayesian filter using Monte Carlo sampling. If $\left\{x_{1:k}^{i},\;i=1,\ldots ,N_{s}\right\}$ is the i-th weighted particle with the i-th weight, $\left\{w_{k|k}^{i},\;i=1,\ldots ,N_{s}\right\}$, the time propagation equation of the PF is as follows:

(8) $$w_{k|k-1}^{i}=p\left(x_{1:k}^{i}|Y_{k-1}\right)=p\left(x_{k}^{i}|x_{1:k-1}^{i},\;Y_{k-1}\right)p\left(x_{1:k-1}^{i}|Y_{k-1}\right)=p\left(x_{k}^{i}|x_{k-1}^{i}\right)w_{k-1|k-1}^{i},$$

$N_{s}$ is the number of the sampled particles and $x_{1:k}^{i}=\left\{x_{1}^{i},\;x_{2}^{i},\;\cdots ,\;x_{k}^{i}\right\}$. If $x_{k}^{i}∼q\left(x_{1:k}|Y_{k-1}\right)$, $i=1,\cdots ,N_{s}$ is a sample generated from the target probability density, $q\left(x_{1:k}|Y_{k}\right)$, the above weight is as follows by the importance sampling principle:

The process of PF can be expressed as follows by separating the time propagation equation and the measurement update equation:

(9) $$x_{k}^{i}∼q\left(x_{k}^{i}|x_{k-1}^{i},\;y_{k}\right),$$

(10) $$w_{k|k-1\;}^{i}=p\left(x_{k}^{i}|x_{k-1}^{i}\right)w_{k-1|k-1}^{i},$$

(11) $$w_{k|k\;}^{i}=\frac{p\left(y_{k}|x_{k}^{i}\right)}{\sum_{i=1}^{N_{s}}p\left(y_{k}|x_{k}^{i}\right)w_{k|k-1}^{i}}w_{k|k-1}^{i},$$

In this study, the current value of the state vector is determined by the one previous value that uses a Markov process, and the Gaussian distribution is used as the target distribution, $q\left(x_{k}^{i}|x_{k-1}^{i},\;y_{k}\right)$. By using the Dirac delta function, the posterior pdf, $p\left(x_{k}|Y_{k}\right)$ at the k step can be approximated as (12):

(12) $$p\left(x_{k}|Y_{k}\right)\approx \sum_{i=1}^{N_{s}}w_{k|k}^{i}\delta \left(x_{k}-x_{k}^{i}\right),$$

Here, δ denotes the Dirac delta function. The state variable estimate and covariance that minimize the mean square error are as follows [8]:

(13) $${\hat{x}}_{k|k}\approx \sum_{i=1}^{N_{s}}w_{k|k}^{i}x_{k}^{i},$$

(14) $${\hat{p}}_{k|k\;}\approx \overset{N_{s}}{\underset{i=1}{\sum }}w_{k|k}^{i}\left(x_{k}^{i}-{\hat{x}}_{k|k}\right){\left(x_{k}^{i}-{\hat{x}}_{k|k}\right)}^{T},$$

The SIS-PF updates the weights and particles when the measurements are put sequentially. When the method runs several steps, a degeneracy problem occurs in which the weights of all particles are too small, except for a few particles. To solve this problem, the number of valid samples, $N_{eff}$, should be maintained [28]. $N_{eff}$ is determined by the user and is set to $\frac{2}{3}N_{s}$ in this study:

(15) $$N_{eff}=\frac{N_{s}}{1+Var\left(w_{k}^{*i}\right)}∼\frac{1}{\sum_{i=1}^{N_{s}}{\left(w_{k}^{i}\right)}^{2}}\leq N_{s},$$

Here, $w_{k+1|k}^{*i}=\frac{p\left(x_{k+1}^{i}|Y_{k}\right)}{q\left(x_{k+1}^{i}|x_{k},\;y_{k+1}\right)}$. It is hard to calculate the target weight, $w_{k+1|k}^{*i}$, as the target distribution is unknown exactly. So, the estimate is used as shown in the above equation. That is, to maximize $N_{eff}$, important sampling is performed so that $Var\left(w_{k}^{*i}\right)$ becomes the minimum. The simplest way to implement this is to perform resampling, and this PF is called sequential importance resampling-PF (SIR-PF). Among various resampling methods, this study employed the stratified sampling method with simulation. This method is as follows [28]:

(16) $$N_{eff}\left[{\left\{x_{k}^{n}\right\}}_{n=1}^{N}\right]=Resample\left[{\left\{x_{k}^{i},\;w_{k|k}^{i}\right\}}_{i=1}^{N_{s}},\;N\right],$$

When the number of particles after resampling is N, the weights are recalculated as follows:

(17) $$w_{k|k\;}^{i}=\frac{1}{N}\;,$$

Resampling can resolve the degeneracy problem, but since the particles with large weights are replicated when the filter is updated, a sampling impoverishment problem occurs where the diversity disappears over time. To alleviate this problem, the Markov Chain Monte Carlo (MCMC)-step was added to PF by replacing only particles that satisfy the diversity judgement condition through Metropolis-Hasting sampling [27]:

(18) $${\tilde{x}}_{k+1}^{i}=x_{k+1}^{i}+\epsilon_{k+1}^{i},\;\epsilon_{k+1\;}^{i}∼N\left(\mu ,\;R_{\epsilon \epsilon }\right)$$

Here, μ and $R_{\epsilon \epsilon }$ are determined by considering the move step size to a new set of particles that use the following random walk model where it was set to 0 and 0.002, respectively, in this study. This MCMC-step is performed after the resampling step. The corresponding acceptance probability is expressed as:

(19) $$A\left({\tilde{x}}_{k+1}^{i},\;x_{k+1}^{i}\right)=\min\left\{\frac{p\left({\tilde{x}}_{k+1},\;x_{1:k}|Y_{k+1}\right)\times q\left({\tilde{x}}_{k+1}|x_{k}^{i},\;y_{k+1}\right)}{p\left(x_{k+1},\;x_{1:k}|Y_{k+1}\right)\times q\left(x_{k+1}|x_{k}^{i},\;y_{k+1}\right)},\;1\right\},$$

(20) $${\hat{x}}_{k+1\;}^{i}=\{\begin{array}{c} {\tilde{x}}_{k+1}^{i}\;\mathrm{if}\;U\left(0,\;1\right)<A\left({\tilde{x}}_{k+1}^{i},\;x_{k+1}^{i}\right)\\ x_{k+1}^{i}\;\mathrm{otherwise} \end{array},$$

To increase the accuracy of the posterior pdf estimate, the number of particles must increase. As the dimension of the state variable increases, the amount of computation increases rapidly [28]. To solve this problem, there have been studies that used the marginalization method for efficient computation in the positioning, navigation, and tracking problems [10,11,29]. This is a method that separates the state variables into linear and nonlinear parts. It also applies nonlinear parts to PF and linear parts to construct one KF for each particle. The most general model about RBPF is as follows [9]:

(21) $$x_{k}^{n}=f_{k-1}^{n}\left(x_{k-1}^{n}\right)+F_{k-1}^{n}\left(x_{k-1}^{n}\right)x_{k-1}^{l}+w_{k-1}^{n},$$

(22) $$x_{k}^{l}=f_{k-1\;}^{l}\left(x_{k-1}^{n}\right)+F_{k-1}^{l}\left(x_{k-1}^{n}\right)x_{k-1}^{l}+w_{k-1}^{l},$$

(23) $$y_{k}=h_{k}\left(x_{k}^{n}\;\right)+H_{k}\left(x_{k}^{n}\right)x_{k}^{l}+v_{k},$$

Here, $x_{k}=\left[\begin{array}{c} x_{k}^{n}\\ x_{k}^{l} \end{array}\right]$, $w_{k}=\left[\begin{array}{c} w_{k}^{n}\\ w_{k}^{l} \end{array}\right]∼N\left(0,\;Q_{k}\right)$ and $Q_{k}=\left[\begin{array}{cc} Q_{k}^{n} & Q_{k}^{nl}\\ Q_{k}^{{nl}^{T}} & Q_{k}^{l} \end{array}\right]∼\left[\begin{array}{cc} Q_{k}^{n} & 0\\ 0 & Q_{k}^{l} \end{array}\right]$. The following includes a general formula that consists of the linear state variables of RBPF, $x_{k}^{l}$ and nonlinear state variables, $x_{k}^{n}$ [9,10]. $f_{k-1}^{n}\left(x_{k-1}^{n}\right)$ is the dynamic function of the nonlinear state variables and is equal to $x_{k-1}+u_{k}$ in Equation (1). $F_{k-1}^{n}\left(x_{k-1}^{n}\right)$ is the dynamic function of the nonlinear state variables and determined by the linear state variable in one previous time. It was set to zero matrix in this study. This means that the prediction of the nonlinear state variables is not affected by the linear state variable [9]. $f_{k-1}^{l}\left(x_{k-1}^{n}\right)$ is the dynamic model of the linear state variable determined by the nonlinear state variables. $F_{k-1}^{l}\left(x_{k-1}^{n}\right)$ is the dynamic function of the nonlinear state variable by the linear state variables in one previous time. $H_{k}\left(x_{k}^{n}\right)$ is the measurement model determined by the linear state variable. Assume that $x_{k}^{l}$ follows the normal distribution in the condition given $x_{k}^{n}$ in the above model, the model can be expressed as:

(24) $$x_{k}^{n}-f_{k-1}^{n}\left(x_{k-1}^{n}\right)=z_{k-1}=F_{k-1}^{n}\left(x_{k-1}^{n}\right)x_{k-1}^{l}+w_{k-1}^{n},$$

(25) $$x_{k}^{l}=F_{k-1\;}^{l}\left(x_{k-1}^{n}\right)x_{k-1}^{l}+x_{k-1}^{l}+u_{k}^{l}+w_{k-1}^{l},$$

(26) $$y_{k}-h_{k}\left(x_{k-1\;}^{n}\right)=y_{k}^{\prime }=H_{k}\left(x_{k}^{n}\right)x_{k}^{l}+v_{k},$$

That is, (24) and (26) are the measurement models and (25) is the system model from the viewpoint of $x_{k}^{l}$. In Equation (24), $x_{k}^{n}-f_{k-1}^{n}\left(x_{k-1}^{n}\right)$ is equal to $x_{k}-x_{k-1}-u_{k}$ in Equation (1). Therefore, it is possible to interpret $z_{k-1}$ as a measurement and $w_{k-1}^{n}$ as the corresponding measurement noise from the viewpoint of $x_{k}^{l}$. From the viewpoint of $x_{k}^{n}$, $F_{k-1}^{n}\left(x_{k-1}^{n}\right)x_{k-1}^{l}$ of (25) and $H_{k}\left(x_{k}^{n}\right)x_{k}^{l}$ of (26) are regarded as additional process and measurement noise, respectively. First, $\left(x_{k}^{n,\left[i\right]},\;w_{k|k-1}^{\left[i\right]}\right)$ is calculated through (9) and (10) for $x_{k}^{n}$. By Bayes’ rule, the joint pdf $x_{k}^{l}$ and $x_{k}^{n}$ in the condition of given $Y_{k}=\left\{y_{1},\ldots ,y_{k}\right\}$ is as follows [9]:

(27) $$p\left(x_{k}^{l},\;x_{k}^{n}|Y_{k}\right)=p\left(x_{k}^{l}|x_{k}^{n},\;Y_{k}\right)p\left(X_{k}^{n}|Y_{k}\right),$$

Here, $p\left(x_{k}^{l}|x_{k}^{n},\;Y_{k}\right)$ is analytically tractable and given by the optimal KF. $p\left(x_{k}^{n}|Y_{k}\right)$ can be estimated by PF. ${\hat{x}}_{k|k-1}^{l}$ is calculated by the time propagation of $x_{k-1}^{l}$. Then the conditional pdf for ${\hat{x}}_{k|k-1}^{l}$ is given by applying two-step measurement updates using $z_{k-1}=x_{k}^{n}-f_{k-1}^{n}\left(x_{k-1}^{n}\right)$, where $x_{k}^{n}$ is the value in the time propagation step of PF in the condition of given $x_{k|k-1}^{l}$ and $y_{k}-h_{k}\left(x_{k}^{n}\right)$, where $x_{k}^{n}$ is the value in the measurement update step of PF in the condition of given $x_{k|k}^{l}$:

(28) $$p\left(x_{k}^{l}|x_{k}^{n},\;Y_{k-1}\right)=N_{x_{k}^{l}}\left({\hat{x}}_{k|k-1}^{l},\;P_{k|k-1}^{l}\right),$$

$\left(x_{k|k}^{n,\left[i\right]},\;w_{k|k}^{\left[i\right]}\right)$ is calculated by performing a measurement update through (12) for $x_{k}^{n}$. When $N_{eff}$ is smaller than the threshold value, resampling is performed. Afterwards, Equation (28) is calculated by the measurement update for $x_{k}^{l}$. Finally, the posterior pdf is obtained as follows:

(29) $$p\left(x_{k}^{n}|Y_{k}\right)≅\sum_{i=1}^{N_{s}}w_{k|k}^{\left[i\right]}\delta \left(x_{k}^{n}-x_{k}^{n,\left[i\right]}\right),$$

(30) $$p\left(x_{k}^{l},\;x_{k}^{n}|Y_{k}\right)≅\sum_{i=1}^{N_{s}}w_{k|k}^{\left[i\right]}N_{x_{k}^{l}}\left({\hat{x}}_{k|k}^{l},\;P_{k|k}^{l}\right),$$

$x_{k}^{l}$ is a one-dimensional state vector that is given in terms of altitude error. The time propagation of the linear component of the states and covariance is as follows:

(31) $${\hat{x}}_{k|k-1}^{l}=\sum_{i=1}^{N_{s}}w_{k|k-1}^{\left[i\right]}x_{k-1|k-1}^{l,\left[i\right]},$$

(32) $${\hat{P}}_{k|k-1\;}^{l}={\hat{P}}_{k-1|k-1}^{l}+Q_{k}^{l},$$

The likelihood of the nonlinear part of RBPF is compensated by this estimated altitude error state and covariance:

(33) $$p\left(y_{k}|x_{k}^{n}\right)=p_{v_{k}+\sqrt{p_{k|k-1}^{l}}}\left(y_{k}-h_{k}\left(x_{k}^{n}\right)-x_{k|k-1}^{l}\right),$$

Kalman gain is updated as follows:

(34) $$K_{k}=\frac{{\hat{P}}_{k|k-1}^{l}}{{\hat{P}}_{k|k-1}^{l}+R_{k}},$$

If the measurement is available, the update state and covariance are performed as follows:

(35) $$x_{k|k}^{l,\left[i\right]}=x_{k|k-1}^{l,\left[i\right]}+K_{k}\left[y_{k}-h_{k}\left(x_{k}^{n,\left[i\right]}\right)-x_{k|k-1}^{l,\left[i\right]}\right],$$

(36) $${\hat{P}}_{k|k\;}^{l}=\left(1-K_{k}\right){\hat{P}}_{k|k-1}^{l},$$

(37) $${\hat{x}}_{k|k}^{l}=\sum_{i=1}^{N_{s}}w_{k|k}^{\left[i\right]}x_{k|k-1}^{l,\left[i\right]},\;$$

## 3. Validity Check Logic of Terrain for RBPF Based TAN

**Figure 1:**
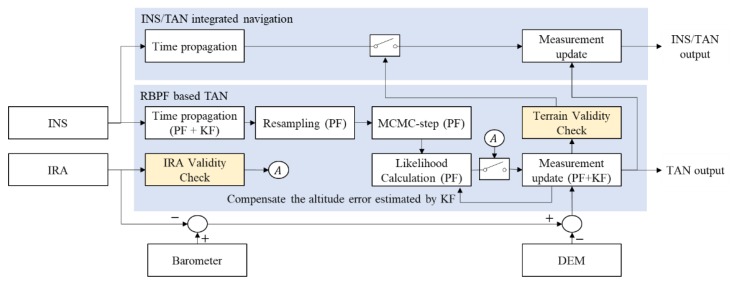
Schematic diagram of the RBPF-based TAN system, including IRA and terrain validity check logic.

In TAN, the validity check technique of measurements by terrain roughness and uniqueness is an important technique that determines the navigation performance. In this study, the interferometric radar altimeter (IRA) is used to measure the angle of the direction of flight, the angle perpendicular to the direction of flight, and the range from the aircraft to the nearest terrain point. It then converts these measurements to a three-dimensional position information on an earth-centered earth-fixed (ECEF) coordinate system [30,31]. Moreover, it can acquire precise position estimates and maintains a very small margin of error, even at high altitudes. Despite these advantages, IRA has many uncertainties, including environmental factors and IRA inherent measurement errors. Generally, the uncertainties are large in flat and repetitive terrains. In particular, it is difficult to estimate the ambiguity errors generated through the signal processing and the glint errors caused by the target fluctuation or clutter, making it challenging to find appropriate compensation techniques. Accordingly, as the TAN that uses the raw data of IRA is likely to be diverted due to uncertain measurements, only the measurements that are useful for TAN should be used selectively. This study describes the RBPF-based TAN, including the validity check logic of the terrain and IRA measurements, as shown in Figure 1. The difference between altitude from the aircraft to the mean sea level (MSL) measured by the barometer and distance from the aircraft to the nearest terrain point measured by IRA was matched with the terrain height on DEM. If the IRA measurement errors are large, PF may not converge. Therefore, this study designed a system that only updates RBPF when it decides the measurement is valid, and if not, the system only conducts time propagation. The INS/TAN integrated navigation uses the estimated position by RBPF-based TAN as measurement and only updates in terrains that seem to be rough and unique through a terrain validity check, as in Figure 1. In this study, we designed an RBPF composed of two-dimensional PF and one-dimensional KF. Two-dimensional PF estimates the latitude and longitude errors. One-dimensional KF estimates the altitude error and compensates the errors in the likelihood step, as shown in Figure 1. If the posterior pdf of the RBPF satisfies the IRA validity check conditions, the IRA measurements are updated. Also, if the posterior pdf is more informative than the prior pdf, the TAN output is judged as satisfying the TAN validity check condition and can be used as the measurements of the INS/TAN integrated navigation.

We need navigation information, including the position, velocity, and attitude. To acquire all the information, increasing the dimensions of RBPF causes computation complexity. There have been studies about the INS/TAN integrated navigation algorithms [32]. In this study, the loosely-coupled INS/TAN integrated navigation was designed to reduce the computing load. The INS/TAN integrated navigation is designed with the EKF and uses the 13th state variables composed with the error of latitude, δϕ, longitude, δλ, velocity, $\left\{\begin{array}{cc} \delta V_{e} & \delta V_{n} \end{array}\right\}$, attitude, $\left\{\begin{array}{ccc} \delta \Psi_{e} & \delta \Psi_{n} & \delta \Psi_{u} \end{array}\right\}$, accelerometer bias, $\left\{\begin{array}{ccc} \delta B_{x}^{a} & \delta B_{y}^{a} & \delta B_{z}^{a} \end{array}\right\}$, and gyro bias, $\left\{\begin{array}{ccc} \delta B_{x}^{w} & \delta B_{y}^{w} & \delta B_{z}^{w} \end{array}\right\}$. Since the TAN filter can be unstable in flat and repetitive terrains, in this study, a feedforward structure was designed to prevent this problem. The state variables can be expressed as:

(38) $$x\left(k\right)={\left[\delta \phi \;\;\delta \lambda \;\;\delta V_{e}\;\;\delta V_{n}\;\delta \Psi_{e}\;\delta \Psi_{n}\;\delta \Psi_{u}\;\delta B_{x}^{a}\;\delta B_{y}^{a}\;\delta B_{z}^{a}\;\;\delta B_{x}^{w}\;\delta B_{y}^{w}\;\delta B_{z}^{w}\right]}^{T},$$

The system and measurement matrix of the discretized state equation are as follows. The system matrix is derived as an error model of INS, and the measurements are acquired from the estimates of the latitude and longitude of the RBPF-based TAN:

(39) x(k)≅(I+AΔt)x(k−1)+w(k−1),

(40) z(k) =Hx(k)+v(k),

(41) Φ(k) =I+AΔt,

Here, $w\left(k-1\right)∼N\left(0,\;Q_{k-1}\right)$, $H=\left[\begin{array}{ccc} 1 & 0 & 0\left[1\times 11\right]\\ 0 & 1 & 0\left[1\times 11\right] \end{array}\right]$, and $v\left(k\right)∼N\left(0,\;R_{k}\right)$.

The system matrix, A is described in Appendix A.

### 3.1. Measurement Validity Check Logic

**Figure 2:**
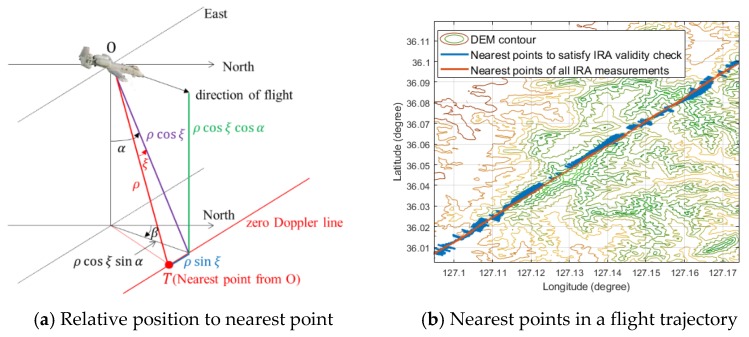
(**a**) Relative position from aircraft to the nearest point and (**b**) nearest points with or without satisfaction of the IRA validity check conditions.

As previously stated, the IRA measurements can be converted into three-dimensional position information. As shown in Figure 2., the relative position vector, $\delta x_{IRA}$, of the nearest point from the aircraft is given in Equation (42):

(42) $$\delta x_{IRA}=\left[\begin{array}{c} \delta \lambda_{res}\\ \delta \phi_{res}\\ h_{res} \end{array}\right]=\left[\begin{array}{c} \rho \cos\xi \sin\alpha \sin\beta +\rho \sin\xi \cos\beta \\ \rho \cos\xi \sin\alpha \cos\beta -\rho \sin\xi \sin\beta \\ \rho \cos\xi \cos\alpha \end{array}\right],$$

Here, ρ and ξ are the range and look angle output of IRA, respectively. The virtual pitch angle, α, and azimuth angle, β, of the zero Doppler line are determined by the velocity of the aircraft as follows [30]:

(43) $$\alpha =\tan^{-1}\frac{V_{u}}{\sqrt{V_{e}^{2}+V_{n}^{2}}},$$

(44) $$\beta =\{\begin{array}{c} \frac{\pi }{2}-{\tan\;}^{-1}\frac{V_{n}}{V_{e}}\;\left|\frac{V_{e}}{V_{n}}\right|>1\\ \tan^{-1}\frac{V_{e}}{V_{n}}\;\left|\begin{array}{c} V_{e}\\ V_{n} \end{array}\right|\leq 1 \end{array},$$

Here, $\left[\begin{array}{ccc} V_{e} & V_{n} & V_{u} \end{array}\right]$ is the velocity of the aircraft in the navigation frame. So, the nearest point, ${\left[\begin{array}{cc} {\hat{x}}_{\phi } & {\hat{x}}_{\lambda } \end{array}\right]}^{T}$, is determined by the summation of the estimated aircraft position calculated by Equation (13) and the relative position, ${\left[\begin{array}{cc} \delta \phi_{res} & \delta \lambda_{res} \end{array}\right]}^{T}$. As shown in Figure 2b, the nearest points acquired by the raw data of IRA measurements without an IRA validity check is very unstable. Therefore, to implement the robust TAN, we must use the beneficial measurements selectively. We could not find the references about the IRA validity check logic for the Bayesian filters. So, we developed a validity check logic through the simulations and captive flight tests.

In this study, the IRA validity check technology was applied using residual check logic in the following equations:

(45) $$\left|\hat{h}-h_{res}-{\hat{x}}_{h}-{\bar{h}}_{dem}\right|<\sqrt{\bar{h_{dem}^{2}}-{\bar{h}}_{dem}^{2}+R+P_{h}},$$

(46) $$\min\left[\hat{h}-h_{res\;}-x_{h}^{\left[i\right]}-h_{dem}\left(x_{\phi }^{\left[i\right]},\;x_{\lambda }^{\left[i\right]}\right)\right]<0.1\times \sqrt{R+P_{h}},$$

Here, $\hat{h}$ is the MSL altitude measured by the barometer. $h_{res}$ is the relative height calculated by the IRA. ${\hat{x}}_{h}$ is the height error estimated by the KF part of RBPF and is equal to the ${\hat{x}}_{k|k}^{l}$ calculated in Equation (37). $x_{h}^{\left[i\right]}$ is equal to $w_{k|k}^{\left[i\right]}x_{k|k-1}^{l,\left[i\right]}$ in Equation (37) and means the estimate of the height error state assigned to the i-th particle. $\left(x_{\phi }^{\left[i\right]},\;x_{\lambda }^{\left[i\right]}\right)$ is the estimate of the latitude and longitude of the i-th particle. ${\bar{h}}_{dem}$ is the mean of the terrain DEM data of the particles and $h_{dem}^{2}$ is the mean of the terrain DEM data squares of the particles. R is the variance of the measurement noise, and $P_{h}$ is the covariance of the height error state estimated by the KF. $\hat{h}-h_{res}$ means the terrain height by the measurements, and ${\hat{x}}_{h}-{\bar{h}}_{dem}$ means the estimate of the terrain height by RBPF. So, the difference between both terms is the residual. $R+P_{h}$ is the acceptable range of the height error square that considers the measurement noise and height error state covariance. $\bar{h_{dem}^{2}}-{\bar{h}}_{dem}^{2}$ is the additional range of the height error square that considers the terrain roughness and uniqueness in the distributed area of particles, so the IRA measurements are valid, but only if the residual is more than one sigma of the acceptable error range, and the minimum residual among the residuals of all the particles is more than 0.1 sigma of the estimated error range by RBPF. These logics were designed through simulations and verified by the captive flight tests.

### 3.2. Terrain Validity Check

As mentioned above, unlike contour matching or BKF based TAN, we could not find a well-established terrain validity check logic in PF based TAN. In this study, a technique was performed that uses mutual information, which is a measure of the mutual dependence between the entropy of a prior distribution and a posterior distribution [33]. Entropy is an index that displays the uncertainties of random variables. If the random variables are in a uniform distribution, the value of entropy is at its maximum. The entropy of the prior pdf, $H\left(x_{k}|Y_{k-1}\right)$ is expressed in terms of the probability $p\left(x_{1:k}^{i}|Y_{k-1}\right)$ in Equation (8) so that:

(47) $$H\left(x_{k}|Y_{k-1}\right)=-\sum_{i=1}^{N_{s}}p\left(x_{1:k}^{i}|Y_{k-1}\right)\log p\left(x_{1:k}^{i}|Y_{k-1}\right),$$

The entropy of the posterior pdf, $H\left(x_{k}|Y_{k}\right)$, is defined as follows [33]:

(48) $$H\left(x_{k}|Y_{k}\right)=-\sum_{i=1}^{N_{s}}p\left(x_{k}^{i}|Y_{k}\right)\log p\left(x_{1:k}^{i}|Y_{k}\right),$$

Mutual information indicates the amount of entropy of $x_{k}$ reduced by measuring $y_{k}$. The validity check index, VIE(k), which uses the mutual information of the estimate, can be determined using Equations (8) and (12):

(49) $$VIE\left(k\right)=H\left(x_{k}|Y_{k-1}\right)-H\left(x_{k}|Y_{k}\right),$$

(50) $$VIE(k)\;=-\overset{N_{s}}{\underset{i=1}{\sum }}w_{k|k-1}^{i}\log w_{k|k-1}^{i}+\overset{N_{s}}{\underset{i=1}{\sum }}w_{k|k}^{i}\log w_{k|k}^{i},$$

**Figure 3:**
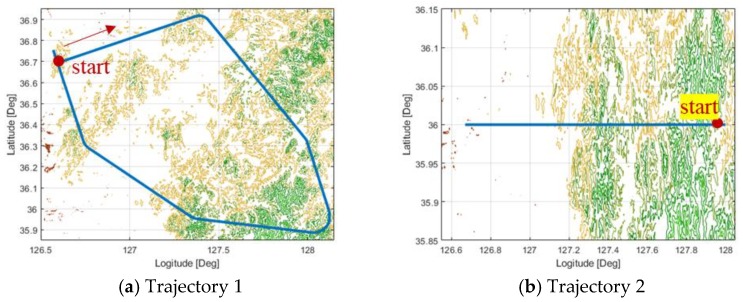

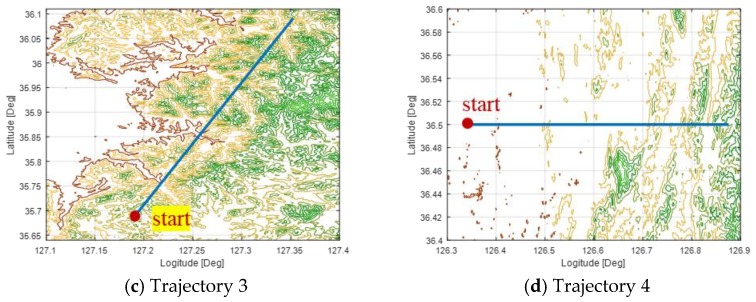
Trajectories for verifying the validity check logic; (**a**) Trajectory 1 starts from an island and includes both flat and rough terrains, (**b**) Trajectory 2 starts from rough terrains and ends at sea, (**c**) Trajectory 3 includes only rough terrains, (**d**) Trajectory 4 starts from flat terrains and ends at rough terrains.

VIE(k) is the amount of reduced uncertainty after the measurement update. In other words, if VIE(k) is positive, the position information estimated by TAN is valid and used as the measurement of the INS/TAN integrated navigation. To verify the method, this study conducted Monte Carlo simulation 100 times based on the simulation condition and RBPF design parameter indicated in Table 1 and Figure 3, respectively. 

Figure 3 shows the simulation trajectories used to observe the performance in various terrain conditions. Figure 3b shows a trajectory that starts from the rough terrain to sea, with Figure 3c showing a trajectory that includes only rough terrains, Figure 3d showing a trajectory that starts from flat land to rough terrain, and Figure 3a showing a trajectory that includes all rough and flat terrains. Through these simulations in various terrains, we want to draw the most optimal design of the validity check logic. This study conducted simulations in various trajectories with VIE and without VIE, and the results are as shown in Table 2. Also, this study compared the navigation error between cases that are decided by VIE(k) in the current point of view and cases that are decided by VIEs accumulated from previous times.

The cases in Table 2 are defined as follows:

- Case 1. RBPF based TAN without IRA and terrain validity check
- Case 2. RBPF based TAN only with IRA validity check
- Case 3. RBPF based TAN with IRA and terrain validity check using VIE(k)
- Case 4. RBPF based TAN with IRA and terrain validity check using VIE(k) and VIE(k−1)
- Case 5. RBPF based TAN with IRA and terrain validity check using VIE(k), VIE(k−1), and VIE(k−2)

As Table 2 indicates, all trajectories had a smaller position error with the IRA and terrain validity check logics than without the check logics. Even if the validity check logic was used, its performance was better on average when the measurement update was conducted with either one of the positive current or one-step previous VIE than if the current VIE was positive, or if either one among the current VIE and the previous two-step VIEs were positive.

Table 2 indicates that the IRA validity check logic provide great improvement. Although the terrain validity check logic is not perfect, it is helpful to improve the performance in some ways. As the IRA validity check logic is a technique to filter out the uncertain IRA measurements caused by the flat and repetitive terrains, a wide sense of the terrain validity check logic is a must for RBPF based TAN. Also, Table 2 shows that the previous data pattern rather than the current data must be considered. Also, these simulation results represent that it is difficult to numerically model the logic helpful to all the common trajectories. So, this study aimed to suggest a design with more improved performance than the conventional RBPF based TAN in various terrains by utilizing deep learning techniques that use time series data, which are called LSTM networks.

## 4. Design of TAN Using RBPF Trained by LSTM Networks

### 4.1. The RBPF Trained by LSTM Networks

**Figure 4:**
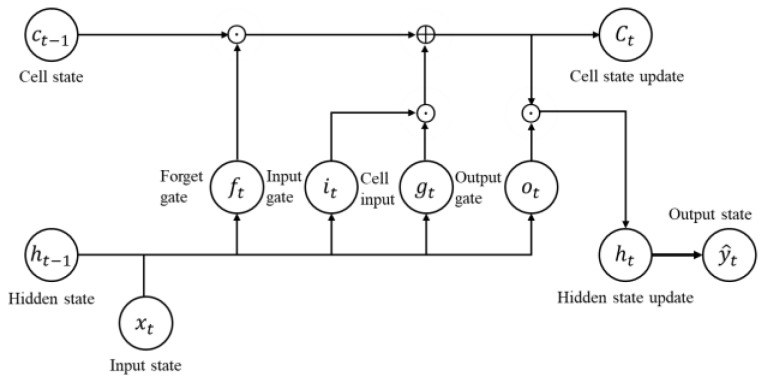
Schematic diagram of a LSTM network.

**Figure 5:**
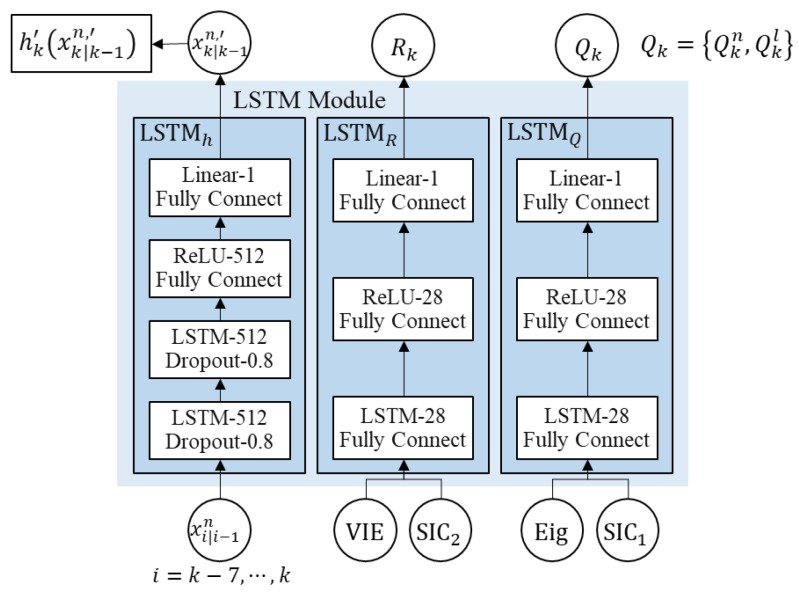
Flow chart of the LSTM module to learn the nonlinear measurement model, process noise covariance, $Q_{k}$, and measurement noise covariance, $R_{k}$.

In the previous section, the two-step validity check logic was designed. The terrain validity check logic was not robust in all the trajectories and was affected by the previous time data of the validity check index. Also, although the IRA validity check logic provided great improvement in those simulations, the incorrect estimates caused the validity check logic to malfunction as the logic was based on the residual between the measurement and the estimated position. Therefore, we proposed the validity check logic using RNN, which is robust in all the terrains and can operate normally in the incorrected estimates. RNN is an artificial neural network that recognizes patterns from time series data. It can memorize patterns from time series data that can consider the current and past input data at the same time. RNN can process various lengths of sequence information, but actually, it only effectively processes comparatively short sequences and cannot remember the incidences from the far past. This is due to the vanishing gradient problem, in which the gradient of the output errors cannot be delivered to the initial layer [34]. In other words, it is inappropriate to make simple RNN to learn using back propagation through time (BPTT) for dealing with longer time series data [31,34]. Among the various tricks for solving the vanishing gradient problem, there are advanced RNN designs, such as the LSTM network, gated recurrent unit (GRU) network and recurrent highway network (RHN) [34]. The LSTM network is composed of cells attached to 3 gates, as in Figure 4, and each gate decides which input value to apply and how much among the current values to forget or output. As shown in Figure 5, the cell state update includes the addition operation. The RNNs’ units are only composed of multiplication operation, but as the LSTM includes addition, it can alleviate the gradient vanish problem [34].

The LSTM network was used with only one hidden layer. The input gate, $i_{t}$, forget gate, $f_{t}$, output gate, $o_{t}$, cell input, $g_{t}$, cell state, $c_{t}$, hidden state, $h_{t}$, and output state, ${\hat{y}}_{t}$, are as follows [24,26]:

(51) $$i_{t}=\sigma \left(W_{ih}h_{t-1}+W_{ix}x_{t}+b_{i}\right),$$

(52) $$f_{t}=\sigma \left(W_{fh\;}h_{t-1}+W_{fx}x_{t}+b_{f}\right),$$

(53) $$o_{t}=\sigma \left(W_{oh\;}h_{t-1}+W_{ox}x_{t}+b_{o}\right),$$

(54) $$g_{t}=\tanh\left(W_{gh\;}h_{t-1}+W_{gx}x_{t}+b_{g}\right),$$

(55) $$c_{t}=f_{t}⊙c_{t-1\;}+i_{t}⊙g_{t},$$

(56) $$h_{t}=o_{t}⊙\tanh\left(c_{t}\;\right),$$

(57) $${\hat{y}}_{t}=W_{yh\;}h_{t}+b_{y},$$

**Figure 6:**
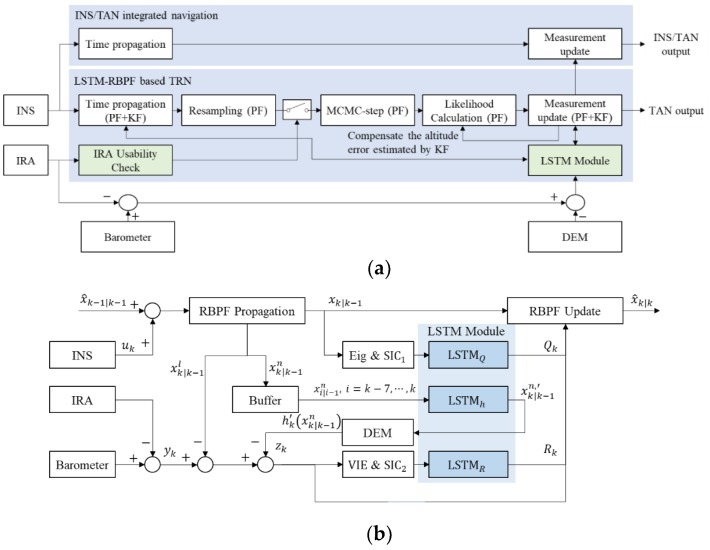
Architecture and detailed block diagram of the proposed TAN using LSTM-RBPF; (**a**) Schematic diagram of the proposed LSTM-RBPF based TAN system, including the IRA measurement availability condition check and LSTM module, (**b**) Block diagram of the proposed RBPF and LSTM networks.

Here, $\left[W_{ih},\;W_{ix},\;W_{fh},\;W_{fx},\;W_{oh},\;W_{ox},\;W_{gh},\;W_{gx},\;W_{yh},\;b_{i},\;b_{f},\;b_{o},\;b_{g},\;b_{y}\right]$ represents the model parameters, including the weighting and bias matrices. σ(·) is an element-wise sigmoid function, and ⊙ denotes the element-wise multiplication of the vectors. The bias of the forget gate was initiated to 1, and the rest of the bias was initiated to 0. All of the initial values of weights were sampled in Gaussian distribution, N(0, 0.1). Figure 6 shows a flow chart of the LSTM module composed of the LSTM layer, rectified linear unit (ReLU) layer, and fully connected linear output layer in Equation (57). ReLU is an activation function defined as the positive part of its argument in the artificial neural networks and is as follows:

(58) $$f\left(x\right)=\{\begin{array}{c} x\;\;\;\;\;\;\;\;\;\;\;\;\mathrm{if}\;x>0\\ 0\;\;\;\;\;\;\;\;\mathrm{otherwise} \end{array},$$

Here, x is the input to a neuron. As it leads to better training results of deeper networks than the logistic sigmoid [35], the ReLU is currently the best used activation function for deep neural networks.

The LSTM module is composed of 4 networks: two ${\mathrm{LSTM}}_{h}$s, ${\mathrm{LSTM}}_{R}$, and ${\mathrm{LSTM}}_{Q}$, as show in Figure 5. Two ${\mathrm{LSTM}}_{h}$s, ${\mathrm{LSTM}}_{R}$, and ${\mathrm{LSTM}}_{Q}$ are the modules to train the $x_{k}={\left[\begin{array}{cc} \delta \phi & \delta \lambda \end{array}\right]}^{T}$ of the measurement model in Equation (2), the measurement noise covariance of the PF part, and the process noise covariance of the PF part, respectively. Both ${\mathrm{LSTM}}_{h}$s have the same structure, which are separately applied to the latitude and longitude errors. At first, this study tried to directly estimate the terrain height, $h_{k}\left(x_{k}\right)$ in the measurement model of Equation (2), but it was impossible to find the patterns of the terrain height. As for the layer composition, the architecture of the proposed LSTM module was designed with the review of Ref. [24], and the number of nodes was tuned by checking the learning performance. The number following the linear, ReLU, and LSTM layers means the number of neurons (or node). Dropout is a regularization technique that alleviates the overfitting problem in various neural networks. The main idea is to randomly drop the connections from the neural network during training [36]. Fully connect means that all the values of the neurons in the current layer are calculated by using all the neurons of the previous layer. Also, unlike the pose estimate problem from images, as the means and variances for normalization the input are unpredictable in the case of the navigation system, this study used variables through the following equations:

(59) $$Eig=\sqrt{\left\|D\left(1,1\right)V\left(1\right)\|+\|D\left(2,2\right)V\left(2\right)\right\|},$$

(60) $$SIC_{j}=\frac{\sum_{i=1}^{N_{s}}P_{j}^{i}U^{i}}{\sqrt{\sum_{i=1}^{N_{s}}{\left(P_{j}^{i}\right)}^{2}}\sqrt{\sum_{i=1}^{N_{s}}{\left(U^{i}\right)}^{2}}},\;j=1,2,$$

Here, V and D represent, respectively, the eigen value and vector of the estimate of covariance, ${\hat{p}}_{k|k-1}$ in Equation (7). $SIC_{j}$ is a similarity index that uses cosine similarities between the prior probability, $P_{1}^{i}$, or the posterior probability, $P_{2}^{i}$, and the uniform probability, $U^{i}$, of the i-th particle [33]. VIE is the validity check index that uses entropy in Equation (50). When these variables are used as input, there is no need for the normalization step, and as the scales of all input terms are similar, it contributes to stable learning. The inputs for each network are as below. The input states of networks for process and measurement noise covariance were considered for the values of the one previous and current step. This is because, as shown in Table 2 from the previous section, when the influence of the validation check logic for RBPF based TAN was considered with the VIEs of the current and one previous step, it had the best navigation performance. Also, as the input of ${\mathrm{LSTM}}_{h}$, the values of the states of PF part from the 7-step previous time to the current time were used. The number for the input data was determined as the value to maximize the training accuracy. Also, after tuning so that the output of the network was between 0 to 2, this study scaled the range below for use. In the regression problem that uses the neural networks, when the range of outputs is between -1 to 1, or 0 to 2, we can acquire stability and high training accuracy [34]. As for the value over 2 or below 0, this study limited them to the maximum and minimum value of the noise covariance for the stability of the filter:

- Input of ${\mathrm{LSTM}}_{Q}$: $\left[Eig\left(k\right)\;SIC_{1}\left(k\right)\;Eig\left(k-1\right)\;SIC_{1}\left(k-1\right)\right]$
- Input of ${\mathrm{LSTM}}_{R}$: $\left[VIE\left(k\right)\;SIC_{2}\left(k\right)\;Eig\left(k-1\right)\;SIC_{2}\left(k-1\right)\right]$
- Input of ${\mathrm{LSTM}}_{h}$: $x_{j|j-1}^{n}$, j=k−7,…,k
- Output of ${\mathrm{LSTM}}_{Q}$ after postprocessing: $1.0\;\leq \;\sqrt{Q_{k}^{n}}\;\leq \;6.0$, $0.5\leq \;\sqrt{Q_{k}^{l}}\leq \;4.0$
- Output of ${\mathrm{LSTM}}_{R}$ after postprocessing: $10.0\;\leq \;\sqrt{R_{k}}\;\leq \;40.0$

${\mathrm{LSTM}}_{h}$ consists of 2 stacked LSTM layers with 512 nodes each, followed by 2 fully connected layers with 512 (ReLU layer) and 1 (linear regression layer) nodes. The input elements of the LSTM layer were randomly dropped in 0.8 probability. This structure uses the same weights, but as learning does not depend on certain neurons or connections, it helps to prevent overfitting. ${\mathrm{LSTM}}_{R}$ and ${\mathrm{LSTM}}_{Q}$ consist of a single layer with 28 hidden states each, followed by 2 fully connected layers with 28 (ReLU layer) and 1 (linear regression layer) nodes. The ReLU function is applied to activate a fully connected layer, except for the last regression layer.

### 4.2. Design of the LSTM-RBPF Based TAN

In Figure 6a, the LSTM module composed of four networks explained in the previous section and the IRA usability check module were used instead of the terrain validity check that uses mutual information and the IRA validity check that uses the residual check method, as shown in Figure 1.

In the IRA usability check module, the conditions are the minimum standards that decide whether TAN can be performed in the current flight state, and the range and look angles that are the outputs of the IRA sensor are received normally. Those conditions are also applied to the conventional RBPF based TAN in the previous section. The usability check conditions are as follows:

- The range output of IRA, ρ>10 m, and the look angle of IRA, $\zeta <10^{{}^{\circ}}$
- The roll angle of aircraft, $\gamma <10^{{}^{\circ}}$, and pitch angle of aircraft, $\varphi <5^{{}^{\circ}}$
- The difference in look angles measured from the left and right antenna, $\left|\Delta \zeta \right|<1^{{}^{\circ}}$

As shown in Figure 6, the proposed method uses more measurement information than the method introduced in the previous section by using the LSTM module that learns the process and measurement noise covariances and the state variation at the measurement update stage. For convenience, the proposed method will be called LSTM-RBPF hereafter. The INS/TAN integrated navigation was designed with the same architecture as the 13th feedforward EKF introduced in the previous section. The block diagram in Figure 6b shows a more detailed flow of the LSTM-RBPF-based TAN. The state estimated in the propagation step, $x_{k|k-1}$, and the estimated state in the previous step that was already stored, $x_{k-1|k-2}$ are converted to input data for ${\mathrm{LSTM}}_{Q}$ through Equations (59) and (60) in the ‘EIG & ${\mathrm{SIC}}_{1}$’ module, as shown in Figure 6b. The outputs of ${\mathrm{LSTM}}_{Q}$ and ${\mathrm{LSTM}}_{R}$ were learned to minimize the loss function, $L_{1}$, calculated by the scaled navigation rms error as shown below:

(61) $$L_{1}=\frac{1}{L_{N}}\sqrt{{\left[\left(\delta \phi_{k|k}-{\hat{\delta \phi }}_{k|k}\right)R_{ns}\right]}^{2}+{\left[\left(\delta \lambda_{k|k}-{\hat{\delta \lambda }}_{k|k}\right)R_{ew}\right]}^{2}},$$

Here, the estimate of state by LSTM-RBPF is ${\hat{x}}_{k|k}={\left[\begin{array}{cc} {\hat{\delta \phi }}_{k|k} & {\hat{\delta \lambda }}_{k|k} \end{array}\right]}^{T}$. $R_{ns}$ and $R_{ew}$ represent the radius of the curvature of the Earth’s ellipsoid in the north-south and east-west, respectively. $L_{N}$ is a scaling factor that limits the extent of the output layer to a specified range and is set to 50 in this study. The output of ${\mathrm{LSTM}}_{h}$ is learned to minimize the loss function, $L_{2}$, as shown below:

(62) $$L_{2}=\frac{1}{L_{N}}\sqrt{{\left[\left(\delta \phi_{k|k}-{\hat{\delta \phi }}_{k|k}\right)R_{ns}\right]}^{2}+{\left[\left(\delta \lambda_{k|k}-{\hat{\delta \lambda }}_{k|k}\right)R_{ew}\right]}^{2}}+\alpha_{reg}\|x_{k|k-1}^{n\;\prime }-x_{k|k-1}^{n}\|{}^{2},$$

Here, $\alpha_{reg}$ is the regularization constant and is set to 0.74. ‖·‖ is the Euclidean distance between the input, $x_{k|k-1}^{n}$, and output, $x_{k|k-1}^{n\;\prime }$. Here, $x_{k|k-1}^{n}={\left[\begin{array}{cc} \delta \phi_{k|k-1} & \delta \lambda_{k|k} \end{array}\right]}^{T}$ is the true value of latitude and longitude that is known. The regularized constant can reduce the overfitting problem. As the result, the proposed ${\mathrm{LSTM}}_{h}$ can provide stable solutions in not only the training data, but also the new test data.

For the training process, there is a need for true noise covariances. But as the true data that can be known represent true position information, it replaced the rms error between the estimated and true noise covariance. The important thing is to tune the range of loss function in order to limit the network output within the desired range. This requires a scaling factor, $L_{N}$, which is set to 50 through numerical simulations. When $L_{N}$ is set to 50, the highest training accuracy is represented. ${\mathrm{LSTM}}_{R}$ estimates the measurement noise covariance in a similar manner. It uses the same loss function, but there is a difference in using VIE and $SIC_{2}$ calculated through Equations (50) and (60) as input data. As for the ${\mathrm{LSTM}}_{h}$, it requires the estimate of state in eight time-steps as the input data, and a buffer was added to store this. Also, the ${\mathrm{LSTM}}_{Q}$ added L2 regularization terms that added the squared difference between the learned state and the estimate of state to loss function.

## 5. Verification of the LSTM-RBPF-Based TAN

### 5.1. Training Accuracy of the LSTM-RBPF

**Figure 7:**
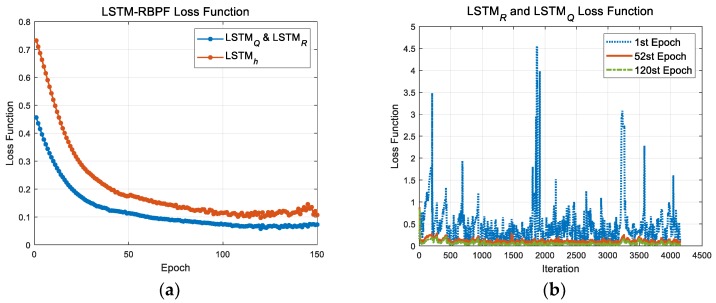
The value of loss function w.r.t. epoch and iteration; (**a**) The values of loss function, $L_{1}$ and $L_{2}$ w.r.t. epoch, (**b**) The values of loss function, $L_{1}$ w.r.t. iteration in the 1st, 52nd, and 120th epochs.

This study used trajectory 1 from Figure 3a for the training data. This trajectory starts from an island and includes sea (flat terrain) and mountains (rough terrain) for 1660 s. We wanted to design a robust validity check logic for any circumstances. So, we used trajectory 1, including all the rough, flat, and repetitive terrains, as a training set. It is important to select the training data properly, as it determines the training and test accuracy. The design and simulation conditions of RBPF were the same as those shown in Table 1. However, the process and measurement noise covariance were used as the initial value until the 8th time-step, and then the learned values in the LSTM module were used. The IRA outputs for the simulation were separate simulator output values, including the signal processing and error model provided by the developer, which the model verified with its similarity with the real outputs through several captive flight tests. The learning parameters and conditions are as shown in Table 3. The maximum epoch was set to 150, and Figure 7a indicates the mean value of the loss function per epoch. One epoch means the duration that all the training data are used to train the model parameters of the LSTM module. One iteration means the duration that the model parameters are updated. In this study, one iteration was performed per 2 Hz. This study used the adaptive moment estimation (Adam) optimizer. As for the optimizer, there are many methods like stochastic gradient descent (SGD) with momentum, adaptive gradient (Adagrad), root mean square propagation (RMSProp), Adam, and more. To speed up the process of training, SGD with momentum memorizes the previously moved direction to the current gradient. Adagrad controls the step size in accordance with the variation of model parameters for the same reason. RMSProp can maintain the difference between the model parameters while preventing the infinite increase of gradient by adding the squared value of the gradient to Adagrad [37]. Adam is the optimizer that builds on the strengths of RMSProp and SGD with momentum, which is defined as below [37]:

(63) $$m_{t}=\beta_{1}m_{t-1}+\left(1-\beta_{1}\right)\nabla_{\theta }L\left(\theta \right),$$

(64) $$v_{t}=\beta_{2}v_{t-1\;}+\left(1-\beta_{2}\right){\left(\nabla_{\theta }L\left(\theta \right)\right)}^{2},$$

(65) $$\theta_{t}=\theta_{t-1\;}-\eta \frac{m_{t}}{\sqrt{v_{t}+\epsilon }},$$

Here, θ is a model parameter such as bias and weight. L(θ) and $\nabla_{\theta }L\left(\theta \right)$ are the values of the loss function, $L_{1}$ or $L_{2}$, and their gradients, respectively. $\beta_{1}$ is the gradient decay factor, and $\beta_{2}$ is the squared gradient decay factor. η is the learning rate, and ϵ is the constant for preventing a divide by zero error and is set to $1\times 10^{-8}$. Generally, when the Adam optimizer is used, the stable training accuracy is acquired quickly. When the magnitude of the gradient of loss function exponentially increase, it is likely that the training becomes instable and diverse in several iterations. The gradient explosion easily occurs in areas where the uncertainties of the measurement output increase or the flat terrain continues. To prevent this, this study used the L2 norm-based gradient clipping method. The gradient threshold was set to 3.4 after considering the error range of the TAN system.

When the epoch is over 120, the value of the loss function of ${\mathrm{LSTM}}_{h}$ becomes rather unstable due to the L2 regularization term. The graph about the value of loss function per iteration in the 1st, 52nd, and 120th epochs is shown in Figure 7b. The significant error due to the uncertainties of measurement and flat terrain in the 1st epoch was confirmed to be significantly improved in the 52nd and 120th epoch. However, when applying the model parameters that passed over 100 epochs to the new test data, there was no improvement effectiveness, which may have been caused by overfitting. In other words, the trained model meets with high training accuracy in the current training data, but doesn’t help to improve the accuracy in the new test data. To prevent this overfitting problem, we stopped the training process in the 52nd epoch, as shown in Figure 7b. In all the simulations below, the learning was performed in an Intel^®^ Xeon^®^, two of CPU @2.10GHz, 64.0GB DDR3 RAM computing environment of thinkstation P900 model (Lenovo, Beijing, China).

**Figure 8:**
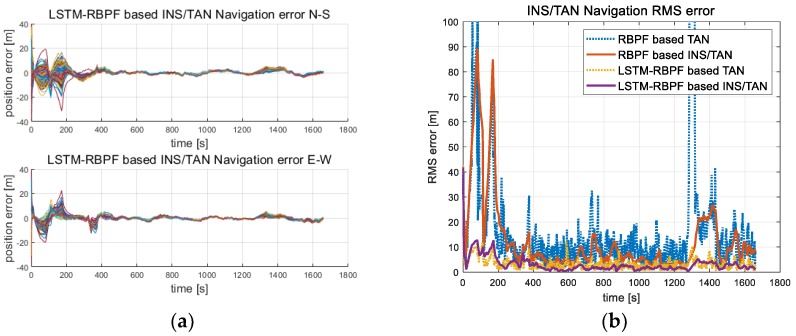
Monte Carlo simulation results of the proposed LSTM-RBPF-based TAN and INS/TAN integrated navigation; (**a**) INS/TAN error in Trajectory 1 [100 times], (**b**) Rms error in Trajectory 1 [seed number = 1].

Figure 8 indicates the results of 100 Monte Carlo simulations where the proposed LSTM-RBPF- based TAN algorithm was performed in trajectory 1 from Figure 3a. When compared with the results of the conventional RBPF based TAN, as shown in Figure 8, it was confirmed to have a stable performance, especially in the sea area.

### 5.2. Evaluation Accuracy of the LSTM-RBPF

**Figure 9:**
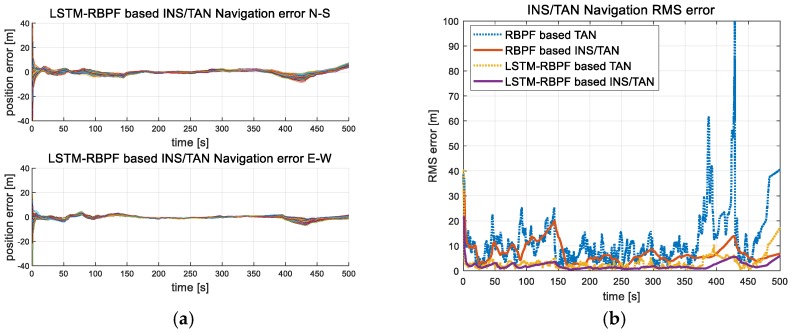

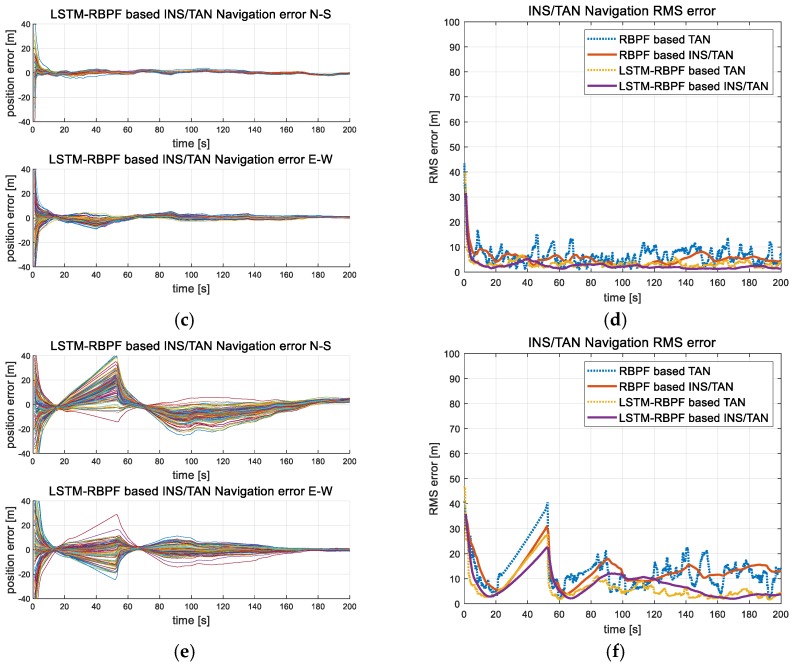
Monte Carlo simulation results of the proposed LSTM-RBPF based TAN and INS/TAN integrated navigation; (**a**) INS/TAN error in Trajectory 2 [100 times], (**b**) Rms error in Trajectory 2 [seed number = 1], (**c**) INS/TAN error in Trajectory 3 [100 times], (**d**) Rms error in Trajectory 3 [seed number = 1], (**e**) INS/TAN error in Trajectory 4 [100 times], (**f**) Rms error in Trajectory 4 [seed number = 1].

To verify the design of the learned LSTM-RBPF, this study applied the design to new test data, not the training data. Figure 9 shows the results of the TAN and INS/TAN integrated navigation errors when the proposed LSTM-RBPF-based TAN performs the Monte Carlo simulation 100 times. When compared with the conventional RBPF-based TAN, it was confirmed that its navigation performance was excellent in all trajectories. In the case of trajectory 2, as it passes to the sea after 350 s, the RBPF-based TAN rms error was significantly diverse. Of course, when the sea continued, the LSTM-RBPF-based TAN eventually becomes diverse, as shown in Figure 9b, but its degree was much less than the RBPF-based TAN. In Table 2, the IRA and terrain validity check logic applied to the conventional RBPF-based TAN was essential to prevent filter divergence in flat and repetitive terrains, but it had an inverse effectiveness in trajectory 3, including only rough terrains. On the other hand, as shown in Figure 9c,d, the performance improved, even in only rough terrain. As for trajectory 4, which started from the sea and ended in rough terrain, the conventional RBPF-based TAN did not perfectly converge, but the proposed method converged quickly when it entered the rough terrain, as shown in Figure 9e,f.

**Figure 10:**
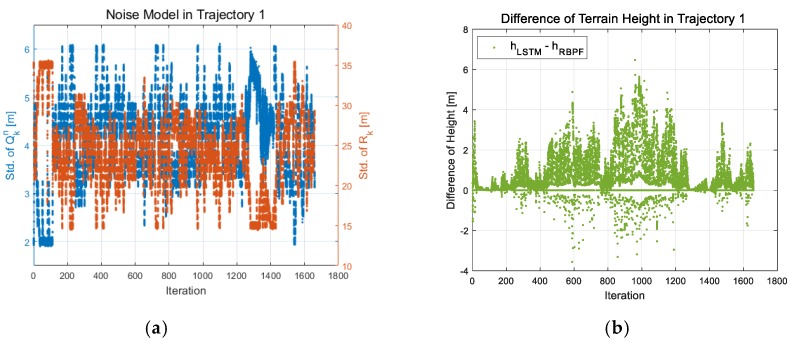

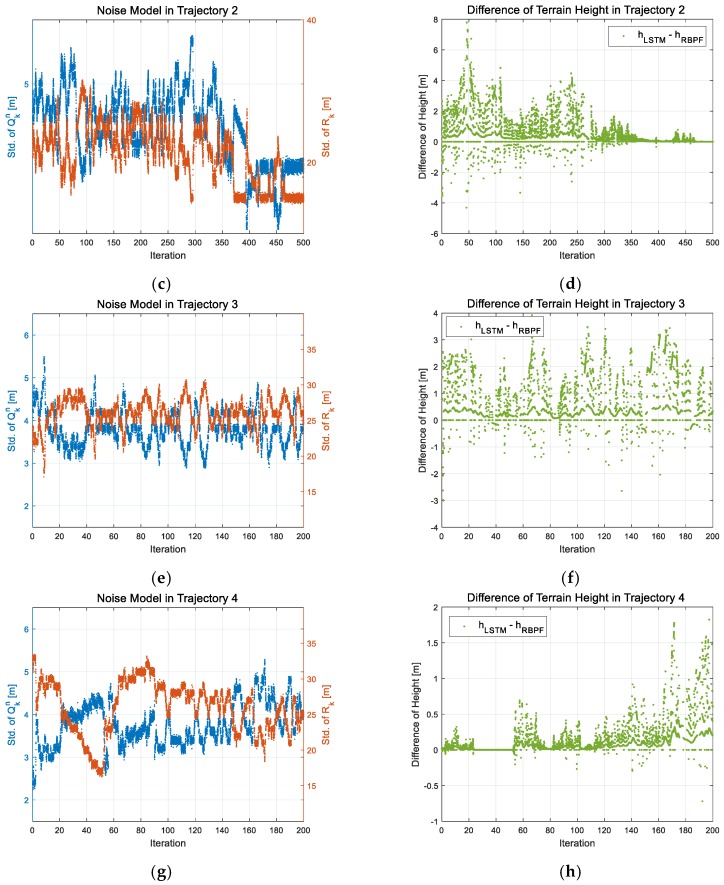
Process and measurement noise covariances of the LSTM-RBPF and difference between terrain heights of the RBPF and the LSTM-RBPF based TAN w.r.t. iteration in the 52th epoch; (**a**) Noise covariances in Trajectory 1, (**b**) Difference of heights in Trajectory 1, (**c**) Noise covariances in Trajectory 2, (**d**) Difference of heights in Trajectory 2, (**e**) Noise covariances in Trajectory 3, (**f**) Difference of heights in Trajectory 3, (**g**) Noise covariances in Trajectory 4, (**h**) Difference of heights in Trajectory 4.

To understand the characteristics of the learned process and measurement noise covariances, the standard deviations of the noise covariances per iteration are shown in Figure 10a,c,e,g. The learned process noise covariance, $Q_{k}$, was 3-dimensional data composed of two $Q_{k}^{n}$ and one $Q_{k}^{l}$. 

In Figure 10, either of $\sqrt{Q_{k}^{n}}$ is shown as the others represent the same characteristics. The process noise in the system model means the reliability of the system, and the measurement noise in the measurement model represents the reliability of the measurement sensor. In the most adaptive filters, the two terms were generally predicted to be in an inverse proportion. This is shown in Figure 10e, which is the result of trajectory 2 with only the rough terrain. However, in the rest of the trajectories, including the sea, there is no similar pattern. As shown in Figure 10a, the process noise gradually decreases in the sea (1200~1500 s), but the measurement noise is maintained at a small value. On the other hand, Figure 10c confirms to have a smaller process and measurement noise after 350 s. Meanwhile, Figure 10g has a smaller measurement noise in the sea (20~60 s), while the process noise is maintained in large values. This is the learning result from reducing navigation errors that cannot be modeled numerically. Also, Figure 10b,d,f,h represent the differences between the terrain height of the new position learned by LSTM-RBPF-based TAN and the height of the position estimated by time propagation in the conventional RBPF-based TAN. As we noted in the previous Section 4, it was impossible to directly estimate the terrain height, $h_{k}\left(x_{k}\right)$, in the measurement model of Equation (2), so two ${\mathrm{LSTM}}_{h}$s were used to train $x_{k}={\left[\begin{array}{cc} \delta \phi & \delta \lambda \end{array}\right]}^{T}$ in Equation (2). To verify the measurement model learned by LSTM-RBPF-based TAN, Figure 10b,d,f,h represent how large is the difference between the terrain height of the conventional RBPF-based TAN and the terrain height at the state, $x_{k}$, learned by the ${\mathrm{LSTM}}_{h}$ module. In the rough terrain, there was an overall 2 m difference, as shown in Figure 10h, but there was also a difference in the maximum 8 m, as shown in Figure 10b,d. As shown in Figure 10d,h, unlike the prediction, there was no difference in the terrain height between the RBPF and the LSTM-RBPF-based TAN in the sea, but it was due to a low altitude of sea terrain. It does not mean there was an insignificant difference between learned and estimated positions. Table 4 below is the result of comparison between the conventional and proposed methods. According to Table 2, Case 4 shows the most stable performance among the conventional RBPF based TAN with various validity check logics. But the navigation performance of the proposed LSTM-RBPF based TAN is better than Case 4 for all trajectories. It was verified that the proposed method showed excellent navigation performance in all trajectories. As for the analysis of the average navigation result, the TAN error of the proposed method was about 37.0% of the conventional RBPF based TAN, and the TAN/INS error of the proposed method was about 47.2% of the RBPF based TAN. The results verified that the proposed method is robust to flat and repetitive terrains and the uncertainties of measurement outputs than the conventional RBPF based TAN.

## 6. Conclusions

This study applied a deep learning method based on LSTM network to improve the performance of TAN that could be replaced in environments where GPS is unavailable. In the case of TAN, it has advantages as it is not affected by external jamming or climate, but its navigation performance degrades when the roughness and uniqueness of the terrain are not secured. Thus, for a highly precise TAN navigation performance, a terrain validity check logic is needed. However, most studies on the TAN technique focused on rough and unique terrains or introduced the method of avoiding flat and repetitive terrains by using the path planning and SLAM techniques. In particular, for the PF-based TAN, there is no verified validity check technique, so, in this study, the terrain and IRA validity check logic by using MI and the residual check method were designed to improve the conventional RBPF- based TAN. However, this study demonstrated that the validity check logic of the conventional RBPF-based TAN for improving navigation performance in flat or repetitive terrains occasionally has an inverse effectiveness in rough terrains through Monte Carlo simulations.

Next, this study proposed the LSTM-RBPF-based TAN that trains the measurement model with strong non-linearity and the process and measurement noise covariances of RBPF to minimize navigation errors. There have been studies that estimated the process and measurement noise. However, the method cannot guarantee stable navigation performance in flat and repetitive terrains. Otherwise, the proposed LSTM-RBPF-based TAN was verified as being able to improve the performance of TAN and INS/TAN integrated navigation in all trajectories, including rough and flat terrains, through Monte Carlo simulations.

We will apply the proposed LSTM-RBPF-based TAN on embedded computing boards and conduct captive flight tests on aircraft in the future. We are currently doing studies about the real-time implementation of the proposed design.

## Figures and Tables

**Table 1:** Simulation conditions and RBPF design parameters.

**Parameter**	**Value**
Initial covariance in PF part	$30^{2}\left[\begin{array}{cc} 1 & 0\\ 0 & 1 \end{array}\right]{\;m}^{2}$
Initial covariance in KF part	$15^{2}m^{2}$
Initial velocity error	[0.1, 0.1, 0.1] m/s
Misalignment angle error	[0.1, 0.1, 1] mrad
Average flight altitude	1 km
Accelerometer bias	100 μg
Gyro bias	0.01 deg/h
Gyro white noise	$0.005\;\deg/\sqrt{h}$
Barometer bias	14 m
Barometer scale factor	0.2% of height
Barometer white noise	5 m
DTED resolution	0.1 arcsec (level 3)
Process noise covariance in PF part	$5^{2}\left[\begin{array}{cc} 1 & 0\\ 0 & 1 \end{array}\right]m^{2}$
Process noise covariance in KF part	$3^{2}{\;m}^{2}$
Measurement noise covariance	$R_{k}=30^{2}\;m^{2}$
Update frequency	50 Hz
Number of particles	1000
Move step parameter, $R_{\epsilon \epsilon }$	0.002

**Table 2:** Simulation results w.r.t. various validity check conditions (unit: mCEP).

Trajectory	Value	Case 1	Case 2	Case 3	Case 4	Case 5
1	TAN	33.106	22.110	22.438	21.943	21.778
	INS/TAN	20.386	15.447	17.058	16.058	15.944
2	TAN	49.514	13.813	17.169	13.346	14.109
	INS/TAN	44.733	6.313	10.024	6.286	6.335
3	TAN	8.371	5.437	6.010	5.964	5.959
	INS/TAN	6.188	4.276	5.121	4.505	4.501
4	TAN	35.755	14.412	14.017	11.923	12.073
	INS/TAN	41.965	13.311	12.998	11.078	11.830
Average	TAN	31.687	13.943	14.909	13.294	13.480
	INS/TAN	28.318	9.937	11.300	9.482	9.653

**Table 3:** Learning parameters and conditions.

Parameter	Value
Maximum epoch	150
Initial bias of LSTM layer	0.0 except for forget bias Forget bias = 1.0
Initial weights of LSTM layer	N(0, 0.1)
Initial bias of fully connected layer	0.0
Initial weights of fully connected layer	N(0, 0.01)
Learning rate	0.005
Drop ratio of learning rate	0.9 times per 20 epochs
Gradient clipping	Norm-based gradient clipping Threshold = 3.4
L2 regularization factor	0.002
Optimizer	Adam
Gradient decay factor	0.9
Squared Gradient decay factor	0.99

**Table 4:** Evaluation simulation results of the proposed LSTM-RBPF based TAN.

Trajectory	Value	Conventional RBPF	Proposed LSTM-RBPF
1	TAN	21.943 mCEP	3.804 mCEP
	INS/TAN	16.058 mCEP	3.956 mCEP
2	TAN	13.346 mCEP	4.162 mCEP
	INS/TAN	6.286 mCEP	2.875 mCEP
3	TAN	5.964 mCEP	2.837 mCEP
	INS/TAN	4.505 mCEP	2.772 mCEP
4	TAN	11.923 mCEP	5.784 mCEP
	INS/TAN	11.078 mCEP	6.013 mCEP
Average	TAN	13.294 mCEP	4.147 mCEP
	INS/TAN	9.482 mCEP	3.904 mCEP

## Appendix A

The system matrix, A is as follows:

(A1) $$A=\left[\begin{array}{cc} F_{1}\left[7\times 7\right] & F_{2}\left[7\times 6\right]\\ 0\left[6\times 7\right] & 0\left[6\times 6\right] \end{array}\right],$$

Here, $F_{2}=\left[\begin{array}{cc} 0\left[2\times 3\right] & 0\left[2\times 3\right]\\ C_{b}^{n}\left[2\times 3\right] & 0\left[2\times 3\right]\\ 0\left[3\times 3\right] & C_{b}^{n}\left[3\times 3\right] \end{array}\right]$. $C_{b}^{n}$ is the coordinate transformation matrix from the body frame to the navigation frame. $F_{1}$ is as follows:

(A2) $$F_{1}=\left[\begin{array}{ccccccc} 0 & F_{12} & F_{13} & 0 & 0 & 0 & 0\\ 0 & F_{22} & 0 & F_{24} & 0 & 0 & 0\\ 0 & F_{32} & F_{33} & F_{34} & 0 & F_{36} & F_{37}\\ 0 & F_{42} & F_{43} & F_{44} & F_{45} & 0 & F_{47}\\ 0 & F_{52} & 0 & F_{54} & 0 & F_{56} & F_{57}\\ 0 & F_{62} & F_{63} & 0 & F_{65} & F_{66} & 0\\ F_{71} & 0 & F_{73} & 0 & F_{75} & F_{76} & 0 \end{array}\right],$$

(A3) $$F_{12}=\frac{V_{e}}{R_{ew}}\tan\phi \sec\phi -\frac{V_{e}}{R_{ew}^{2}}\sec\phi \delta R_{ew},\;F_{13\;}=\frac{1}{R_{ew}}\sec\phi ,$$

(A4) $$F_{22}=-\frac{V_{n}}{R_{ns}^{2}}\delta R_{ns},\;F_{24\;}=\frac{1}{R_{ns}},$$

(A5) $$\;F_{32\;}=2U_{0}\sin\phi V_{u}+2U_{0}\cos\phi V_{n}-\frac{\delta R_{ew}}{R_{ew}}\left(\frac{V_{n}V_{u}}{R_{ns}}+\frac{V_{e}V_{n}}{R_{ew}}\right)+\frac{V_{n}V_{e}}{R_{ew}}\sec^{2}\phi ,\;F_{33}=\frac{\tan\phi V_{n}-V_{u}}{R_{ew}},$$

(A6) $$\;F_{34\;}=\frac{V_{e}}{R_{ew}}\tan\phi +2U_{0}\sin\phi ,\;F_{44}=-\frac{V_{u}}{R_{ns}},$$

(A7) $$\;F_{36\;}=-f_{u},\;F_{37}=f_{n},\;F_{45}=f_{u},\;F_{47}=-f_{e},$$

(A8) $$\;F_{42\;}=-2U_{0}V_{e}\cos\phi -\frac{V_{e}^{2}}{R_{ew}}\sec^{2}\phi +\frac{V_{n}V_{u}}{R_{ns}^{2}}\delta R_{ns}-\frac{V_{e}^{2}}{R_{ew}^{2}}\tan\phi \delta R_{ew},\;F_{43}=-\frac{2V_{e}}{R_{ew}}-2U_{0}\sin\phi ,$$

(A9) $$\;F_{52\;}=\frac{V_{n}}{R_{ns}^{2}}\delta R_{ns},\;F_{54}=-\frac{1}{R_{ns}},\;F_{56}=\frac{U_{0}V_{e}\sin^{2}\phi }{R_{ew}\cos\phi },\;F_{57}=-\frac{U_{0}V_{e}\cos\phi }{R_{ew}},$$

(A10) $$\;F_{62\;}=-\frac{V_{e}}{R_{ew}^{2}}\delta R_{ew}-U_{0}\sin\phi ,\;F_{63}=\frac{1}{R_{ew}},\;F_{65}=-U_{0}\frac{V_{e}\sin^{2}\phi }{R_{ew}\cos\phi },\;F_{66}=-\frac{V_{n}}{R_{ns}},$$

(A11) $$\;F_{71\;}=\frac{V_{e}}{R_{ew}}\sec^{2}\phi -\frac{V_{e}}{R_{ew}^{2}}\cos\phi \delta R_{ew},\;F_{73}=\frac{\tan\phi }{R_{ew}},\;F_{75}=U_{0}\frac{V_{e}}{R_{ew}}\cos\phi ,\;F_{76}=\frac{V_{n}}{R_{ns}},$$

Here, $R_{ew}$ and $R_{ns}$ represent the radius of curvature of the Earth ellipsoid in the east-west and north-south, respectively. The perturbation of these are $\delta R_{ew}$ and $\delta R_{ns}$. $\left[\begin{array}{ccc} V_{e} & V_{n} & V_{u} \end{array}\right]$ is the velocity of the aircraft in the navigation frame. ϕ and λ is the latitude and longitude of the aircraft, respectively. $U_{0}$ is the rotation velocity of the Earth and $\left[\begin{array}{ccc} f_{e} & f_{n} & f_{u} \end{array}\right]$ is the specific force of the accelerometer.
